# Prostate cancer: histopathological analysis of 66 cases

**DOI:** 10.1186/1750-9378-6-S1-A4

**Published:** 2011-08-11

**Authors:** Olabode Peter Oluwole, Abdulrahuaman Umar Murkthar, Chinwuba Amaechina

**Affiliations:** 1 Department of Pathology, College of Health Sciences, University of Abuja, Nigeria; 2 Department of Pathology, University of Abuja Teaching Hospital, Abuja, Nigeria; 3 Department of Surgery, University of Abuja Teaching Hospital, Abuja, Nigeria

## Background

In the United States and Europe, prostate cancer is the most common male malignancy and the second leading cause of cancer death after lung cancer. However, in spite of under-estimation and reporting of cancer burden in Pathology-based cancer registries in the developing countries; prostate cancer is now ranked among the emerging male malignancy.

## Objective

To demonstrate that prostate cancer is common among Nigerian males.

## Materials and methods

This is a 5-year retrospective histopathological analysis of prostate cancer diagnosed between January 2005- December 2009 in the Department of Pathology, University of Abuja Teaching Hospital, Gwagwalada, Abuja. The tumours were classified based on WHO classification of Prostatic tumours. Histological grading was done using Gleason grading system.

## Results

Sixty-six (66) cases of prostate cancer were reviewed. The age range was between 40-89 years with a mean of 64.5 years. There were two bimodal age peaks at the seventh and eighth decades respectively. The surgical procedures were Trucut biopsies 64 (96.9%) and prostatectomy 2 (3.1%). The Gleason scores range from 2 to 10. Three (4.5%) patients were seen before the age of 50 years. There were 9 (13.6%) cases of well differentiated, 37 (56.0%) cases of moderately differentiated and 20 (30.4%) cases of poorly differentiated adenocarcinoma. No other histological types were seen.

## Conclusion

In this study, there were bimodal age peaks of prostate cancer at the sixth and seventh decades, while moderately differentiated adenocarcinoma is the most common presentation.

## Acknowledgements

Publication of this article was funded in part by the University of Florida Open-Access Publishing Fund.

